# Bacteriological and serological investigation of *Clostridium perfringens* in lambs

**DOI:** 10.1038/s41598-022-21918-6

**Published:** 2022-11-16

**Authors:** Shymaa Moustafa, Islam Zakaria, Abdelmoneim Moustafa, Rania AboSakaya, Abdelfattah Selim

**Affiliations:** 1 Department of Animal Medicine (Infectious Diseases), Faculty of Veterinary Medicine, Benha University, Toukh, 13736 Egypt; 2 Animal Health Research Institute, Dokki, Giza, Egypt

**Keywords:** Microbiology, Diseases

## Abstract

*Clostridium perfringens* is one of the most common and important pathogens in livestock due to its ability to produce a diverse arsenal of toxins. Owing to *C. perfringens* economic importance, this study aimed to determine the types and toxins of *C. perfringens* in newly born lambs. A total of 200 lambs of less than one-month old were examined, including 100 lambs suffered from diarrhea, 60 freshly dead and 40 apparent healthy. *C. perfringens* was identified morphologically and biochemically using bacteriological culture in 103 of 200 samples (51.5%). Moreover, serological typing of *C. perfringens* isolates revealed three serotypes, *C. perfringens* type A (54.2%), *C. perfringens* type B (28.8%) and *C. perfringens* type D (16.9%). The highest prevalence rate for *C. perfringens* infection was observed in winter (58.25%) in comparison with other seasons. The findings of the present study confirm the presence of enterotoxmia among lambs in localities under study, causing economic losses. The proper vaccination schedule particularly against *C. perfringens* type A and B is highly recommended.

## Introduction

*Clostridium perfringens* is a Gram-positive, anaerobic bacteria that causes a variety of human and animal diseases^1^. It is frequently discovered in the gastrointestinal system of animals and is widely distributed in the environment (e.g., in soil and sewage)^2^.

*Clostridium perfringens* infections have been associated with severe diseases in young animals that may result in significant economic lossess. These diseases are distinguished by a rapid progression and high mortality rate, as well as symptoms of colic, hemorrhagic enteritis, convulsions, and neurological abnormalities^3^.

*Clostridium perfringens* is classified into five toxinotypes (A, B, C, D, and E) based on the synthesis of four main toxins: *alpha* (α), *beta* (β), *epsilon* (ε), and *iota* (ι). Each toxin type is contributed to particular intestinal diseases in different animal species^4,5^. All toxinotypes generate α-toxin, however type B and C strains produce β-toxin, type B and D strains yield ε-toxin, and type E strains produce the ι-toxin^6–8^. Two more significant toxins produced by *C. perfringens* are the enterotoxin and the β2-toxin, and both have been documented in the past ten years^9^.

All five species of *C. perfringens* can induce enterotoxemia. Different *C. perfringens* biotypes are responsible for various human and animal diseases. Type A strains causes gas gangrene and food poisoning in humans and are frequently detected as a normal component of the intestinal microflora of lambs^10^. In sheep, lambs, calves, piglets, and poultry, type C strains induce enterotoxemia and necrotic enteritis^11,12^. Dysentery and pulpy kidney disease are believed to be caused by type D strains in sheep and lambs^13^.

The usual procedures for isolating, cultivating, and typing *C. perfringens*, such as the mouse neutralization tests, are time-consuming, expensive, and necessitate the use of live animals and monovalent diagnostic sera. In addition to the ethical issues, the mouse neutralization test also has inconsistent and imprecise results^14,15^. ELISA is one of quick and simple methods that are utilized to detect the presence of *C. perfringens* toxins in the intestinal contents of sick animals.

It was thought prudent to verify the ELISA against the more often used method of detection, anaerobic culture using selective media. When applied to nonenriched samples, the ELISA had sensitivity and specificity of 86 and 98%, respectively, and was thus deemed suitable for use in future intergroup comparisons^16^.

It has been discovered that toxin genotyping is more trust worthy than traditional toxin typing. In order to determine the predominant *C. perfringens* types in a given geographic area, toxin genes rather than the poisons they generate must be identified. Classifying the isolates into toxigenic types using colony shape, biochemical characteristics, and measurement of fatty acids and organic acid end products of metabolism by gas–liquid chromatography is challenging^17^.

In Egypt, *C. perfringens* type A, B, and D have been reported in sheep with morbidity rate up to 25% and mortality rate 16.25%^18^. Moreover, Selim, et al.^19^ have been reported *C. perfringens* type A among diarrheic and recently dead calves.

To ensure a thorough understanding of the epidemiology of *C. perfringens* infections, detection of *C. perfringens* toxin types and subtypes is essential. It may also be helpful in the development of an efficient disease control strategy^20^.

Therefore, the present study aimed to determine the prevalence of *C. perfringens* in lambs and to investigate the *C. perfringens* toxino types using ELISA technique.

## Materials and methods

### Ethical approval

The study was performed according to guidelines and regulations of ethical committee of faculty of veterinary medicine, Benha University (BUFVTM29-9-2022). The study was conducting following ARRIVE guidelines.

### Animals and sampling

The study was performed during June 2021–May 2022, to investigate the presence of *C. perfringens* among lambs raised in the Qalyubia and Menofia governorates. A total of 200 samples were randomly collected from lambs (100 fecal samples from lambs had profuse diarrhea, 40 fecal swabs from apparent healthy lambs and 60 intestinal content from recently dead lambs). Samples were placed in sterile, individual polyethylene bags, labelled, and shipped to the lab on ice for examination. The data of each examined lamb including locality, sex and season were reported.

### Isolation and identification of *C. perfringens*

In accordance with previously described procedures of Smith and Holdeman^21^, each sample was inoculated into sterile cooked meat medium and anaerobically incubated at 37 °C for 24–48 h. A loopful from a tube that had been incubated was streaked into 200 g/ml of sheep blood agar for isolation and anaerobically incubated at 37 °C for 24–48 h. The colonies with doubled zone of hemolysis were further identified.

Based on Nagler’s reaction, *C. perfringens* type A alpha antitoxin was inoculated in one half of egg yolk agar plates, and allowed to dry in the incubator for 30 min. From the antitoxin-free half of the plate to the other, the suspected isolated organisms were streaked across it. The plates were evaluated using previously described procedure of Koneman, et al.^22^ after being incubated anaerobically at 37 °C for 24 h., for the appearance of opalescence and formation of pearly layers on the half of the plate without antitoxin.

The collected *C. perfringens* isolates were subjected to biochemical analysis using techniques that had already been reported in previous study of Koneman et al. ^23^. All the isolates were capable of producing H_2_S and reducing maltose, lactose, sucrose, nitrate, and negative for urease reduction.

### Identification of *C. perfringens* toxins

The toxin antitoxin neutralization test was carried out according to Smith and Holdeman^21^. The test was conducted by mixing 0.3 ml of centrifuged supernatant from the cooked meat culture of each kind of *C. perfringens* with 0.1 ml of particular antisera (A, B, C, D, and E).

Additionally, according to the manufacturer’s instructions, the intestinal filtrate and broth culture supernatants of the isolated strains were tested using BIO K 270 - Multiscreen Ag ELISA Enterotoxaemia (Bio-X Diagnostics S.A, Belgium) to identify *C. perfringens* toxins (Alpha, Beta, and Epsilon).

The optical densities (ODs) were measured at 450 nm using a micro plate reader (clindiag MR-96, Belgium).

The measurement of each sample well was subtracted from the OD of the corresponding negative control to determine the net OD for each sample. Any sample that produced a difference in OD 0.150 was regarded as positive for the tested toxins. In accordance with the manufacturer’s QC data sheet, the limit of OD positivity for the alpha, beta, and epsilon toxins is 0.150. On the other hand, a sample was deemed negative if the OD difference was less than 0.150.

### Statistical analysis

All data collected were entered into Microsoft excel spreadsheet. For analysis of the data SPSS version 16 software was used. Data were analyzed descriptively in the first step, using the frequency table and cross tabulation. Then the association of the different variables with the prevalence of *C. perfringens* at the animal level was analyzed using a Chi-square test.

## Results

### Isolation and identification of *C. perfringens*

A total of 103 out of 200 fecal samples (51.5%) were positive. The positive samples for *C. perfringens* showed characteristic features on blood agar like dewdrops, smooth greyish convex colonies with a double zone of hemolysis.

### Prevalence of *C. perfringens* among different examined lambs

The prevalence rate for *C. perfringens* was significantly differed between examined Lambs (*P* < 0.01) and the highest rate was observed among diseased lambs 67% while the lowest rate was observed among apparent healthy lambs 17.5%, Table 1.

**Table 1:** Prevalence of *C. perfringens* in different examined lambs in relation to season.

Seasons	Apparent healthy lambs			Diseased lambs			Dead lambs		
	No of tested lambs	No of positive	%	No of tested lambs	No of positive	%	No of tested lambs	No of positive	%
Summer	9	1	11.11	21	12	57.14	15	8	53.33
Winter	15	5	33.33	47	39	82.97	29	16	55.17
Spring	11	0	0.00	21	7	33.33	7	2	28.57
Autumn	5	1	20	11	9	81.82	9	3	33.33
Total	40	7	17.50	100	67	67	60	29	48.33
	Chi^2^ = 10.45**			Chi^2^ = 12.49**			Chi^2^ = 13.70**		

** = Significant at (*P* < 0.01).

Concerning the seasonal effect on the prevalence of *C. perfringens*, the prevalence rate showed significant disparity (*P* < 0.01) between different seasons. The highest prevalence rate was observed in winter 33.33%, 82.97% and 55.17% in apparent healthy, diseased and dead lambs, respectively in comparison with other seasons, Table 1.

### Typing of *C. perfringens* isolates

The toxigenic *C. perfringens* isolated were examined to determine the type of toxin based on lecithinase activity. The results were significantly differed (*P* < 0.01) between healthy, diseased and different organs of dead lambs, Table 2.

**Table 2:** Typing of *Clostridium perfringens* isolate in guinea pigs.

Animals	Number of toxigenic isolates	Typing							
		A		B		C		D	
		No	%	No	%	No	%	No	%
App. healthy	1	1	100	–	–	–	–	–	–
Diseased animals	38	19	50	13	34.21	–	–	6	15.78
Liver	7	6	85.71	–	–	–	–	1	14.28
Kidney	4	2	50	2	50	–	–	–	–
Intestine	9	4	44.44	2	22.22	–	–	3	33.33
Total	59	32	54.23	17	28.81	–	–	10	16.94
		Chi^2^ = 15.30**		Chi^2^ = 9.40**		–		Chi^2^ = 7.45**	

** = Significant at (*P* < 0.01).

All identified isolates in healthy lambs were *C. perfringens* type A, while type A (50%), B (34.21) and D (15.78%) were investigated in diseased lambs, Table 2. In addition, the *C. perfringnes* type A (85.71%) and type D (14.21%) were identified for isolates from liver while type A and B (50%) were identified in isolates from kidney. However, *C. perfringens* type A (44.44%), B (22.22%) and D (33.33%) were identified among isolates from intestine, Table 2.

### Identification of *C. perfringens* toxins by ELISA

By comparing the results from ELISA, there was a significant difference (*P* < 0.01) regarding type of *C. perfringens* among examined lambs. *C. perfringens* type A was the highest prevalent (43.69%) type, followed by type B (33.99%) and type D (22.33%), Table 3.

**Table 3:** *C. perfringens’* toxins serotyping using ELISA.

Animal	No of tested samples	No of positive samples	*C. perfringens* types				
Lambs	103	103	A	B	C	D	E
			45	35	–	23	–
			43.69	33.99	–	22.33	–
			Chi^2^ = 12.45**				

** = Significant at (*P* < 0.01).

## Discussion

*C. perfringens* is ranked among the most important pathogens in livestock and humans. It causes both histotoxic disease and intestinal infections. Toxins produced by *C. Perfringens* types are responsible for enteric diseases in sheep and goats and it has been suggested that economic losses related to *C. perfringens* infections may be resulted from all seven types of the bacterium^5,24^. It is well known that enterotoxaemia causes considerable economic losses to sheep industry due to a high fatality rate, decreased productivity, and increased treatment costs^5,25^.

Enterotoxemia is one of the most frequently occurring diseases in sheep and goats worldwide. Reports from countries around the world have reported prevalence rates of enterotoxemia ranging between 24.13% and 100% (El Idrissi and Ward, 1992, Greco et al., 2005**)**.

The prevalence rate of *C. perfringens* in the present study was 17.5% among apparent healthy lambs which was lower than previous reported rate (39.71%)^15^, while it was 67% among diseased lambs, which come in line with previous rate (69.29%), reported by Kumar, et al.^15^. Moreover, the overall prevalence rate of *C. perfringens* in different examined lambs based on bacteriological examination was 51.5%. In comparison with the findings of previous studies, we observed the prevalence rate of *C. perfringens* was lower than those reported in Andra Pradesh, India (59.62%)^15^ and higher than those reported in Saudi Arabia (30.41%)^26^ and in Bangladesh (32.1%)^27^.

These prevalence rates may differ due to differences in experimental design, geographic or environmental characteristics, research length, the time of year the investigations were conducted, the use of sanitary precautions, and the diagnostic tests that were utilized^28,29^.

From the present findings, it is clear that the prevalence rate of *C. perfringens* was highest in winter season; it was 33.33%, 82.97% and 55.17% in apparent healthy, diseased and dead lambs, respectively. These findings are proportionally consistent with Selim, et al.^19^, they reported that the higher prevalence rate in winter 95.2% in clinically affected calves and 73.3% in dead calves. The higher incidence in winter may be attributed to poor hygienic conditions and lower temperature. These findings might be contributed to high humidity and changing in pasture during winter season in comparison with other seasons^30^.

Our results demonstrated that, typing of *C. perfringens* by intradermal inoculation test in Guinea pig revealed that the incidence of toxigenic and non-toxigenic strains were 57.3% and 42.7%, respectively. In contrast, Atwa, et al.^31^ found that typing of *C. perfringens* by intradermal inoculation test in albino Guinea pig revealed that the incidence of toxigenic and non-toxigenic strains were 81.9% and 18.1%, respectively in diarrheic calves.

In the current study, *C. perfringens* type A was more prevalent (43.69%), followed by type B (33.99%) and type D (22.33%). This result is closely identical to the finding of Nayel, et al.^18^ who stated that *C. perfringens* type A, B and D are the main types causing diseases in sheep in Egypt, but differed somewhat from a previous study that revealed the predominance of *C. perfringens* type A (67.2%), followed by type D (16.4%), then type B (13.4%) and type C (3%) recovered sheep, goats, cattle and camels in the Kingdom Saudi Arabia.

All *C. perfringens* isolates (n = 103) were examined by multiscreen Ag ELISA to identify type of toxin. The most predominant type was type A (43.68%), followed by type B (33.98%) and type D (22.33%). Regarding the toxin type detected in the tested isolates, types A and B were the dominant types detected by ELISA in lambs. However, previous studies; Fayez, et al.^26^,Moussa and Hessan^32^,Al-Humiany^33^ showed the high prevalence of *C. perfringens* type-A and D among enterotoxaemic sheep.

ELISA has the advantage of being simple and rapid to run, and it can provide additional information about the toxin profile of *C. perfringens* isolates if conditions are favorable for toxin synthesis at the time of sample collection and at a level detectable by the ELISA^16^.

## Conclusion

*C. perfringens* is highly prevalent among lambs. The toxinotype A was more prevalent than toxinotype D in sheep. Absence of toxinotypes C, and E in the present study does not indicate the absence of these toxinotypes in the sheep.

## Data Availability

All data generated or analysed during this study are included in this published article.
